# Endovascular treatment for posterior epistaxis: should it still be considered a last-resort option? A literature review

**DOI:** 10.3389/fsurg.2026.1741949

**Published:** 2026-04-07

**Authors:** Hector R. Martinez, Daniel F. Aguilera-Maldonado, Andrea Paola Sanchez-Cantu, Patricia Maria Orviz-Ortiz, Oscar I. Paz-Sanchez, Nerea Martin del Campo, Carlos Cuilty-Siller, Karla Santos-Santillan, Jorge Arechavaleta Santos, Oscar Gutierrez Trevino, Beatriz Elena Perez-Martinez, Jose A. Figueroa-Sanchez

**Affiliations:** 1 Instituto de Neurologia y Neurocirugia, Centro Medico Zambrano Hellion, TecSalud, San Pedro Garza Garcia, Mexico; 2 Escuela de Medicina y Ciencias de la Salud, Tecnologico de Monterrey, Monterrey, Mexico; 3 Centro Medico Zambrano Hellion, Instituto de Otorrinolaringología, TecSalud, San Pedro Garza Garcia, Mexico

**Keywords:** embolization, endovascular procedures, endovascular therapy, epistaxis, posterior epistaxis

## Abstract

**Introduction:**

Posterior epistaxis is a common otorhinolaryngological emergency that often presents a clinical challenge and typically needs aggressive management. Endovascular therapy has emerged as a strong alternative to traditional approaches. However, it is still considered a last-resort option for intractable cases in most clinical guidelines and among many physicians managing this condition. The purpose of this narrative review is to provide a comprehensive overview of posterior epistaxis, with a particular focus on its vascular anatomy and the role of endovascular treatment.

**Methods:**

Articles were retrieved from MEDLINE, Scopus and Web of Science using a semi-systematic search strategy.

**Results:**

Posterior epistaxis is a challenging condition due to its variable etiologies and numerous risk factors. Effective treatment requires careful consideration of the region's complex and variable vascular anatomy, including potentially dangerous anastomoses. The results indicate that endovascular treatment is a feasible and safe option; however, it should always be preceded by a thorough angiographic assessment to identify the exact vascular anatomy and site of bleeding. This also helps to identify potentially dangerous communications around the skull base, where inadvertent embolization can lead to complications such as stroke or blindness.

**Conclusion:**

Posterior epistaxis is a complex condition that requires a comprehensive approach. Performing an angiography before treatment can help pinpoint the bleeding site, as well as identify any dangerous communications. Endovascular treatment could be a viable and safe option in cases of posterior epistaxis and should not be reserved solely as a last resort.

## Introduction

Epistaxis, or nosebleed, is a common condition that affects 60% of the population at least once in their lifetime, thus making it the most frequent Ear, Nose, and Throat (ENT) emergency consultation (1–7). The severity of epistaxis can vary, ranging from a minor self resolving episode to a rare but life-threatening hemorrhage, depending on the underlying etiology and the volume of bleeding (8, 9). Treatments such as nasal packing, cauterization, endoscopic arterial ligation, and endovascular therapy have been described, each with its benefits and potential complications (7, 10).

Epistaxis is classified into two types based on the location of bleeding within the nasal mucosa: anterior and posterior (11, 12). Anterior epistaxis is more prevalent and usually self resolves after gentle pressure and anterior nasal packing. Posterior epistaxis, on the other hand, is less frequent and often more challenging to manage and typically unresponsive to conservative measures, requiring a more aggressive treatment approach (13). The spectrum of epistaxis severity ranges from mild to potentially fatal bleeding (14). Severe posterior epistaxis is characterized by significant blood loss often requiring nasal packing, and commonly necessitating a jump to more invasive treatments such as cauterization or endoscopic arterial ligation (6, 15, 16).

Anterior and posterior epistaxis are both subclassified based on its resolution status as resolved, recurrent or intractable. Recurrent epistaxis refers to nosebleeds occurring within 30 days after medical intervention intended to achieve hemostasis (5). Intractable epistaxis is defined as nasal bleeding that persists despite conservative measures and requires additional more invasive treatments (11, 13, 17–19). It is important to note that some authors use the terms “posterior epistaxis”, “intractable epistaxis”, and “severe epistaxis” interchangeably (20, 21).

Endovascular treatment for posterior epistaxis, first presented by Sokoloff et al. in 1974, has shown to be an effective option for severe epistaxis cases with a low rate of major complications, making it a strong alternative to commonly used invasive treatments such as cauterization and arterial ligation (13, 20, 22). However, it remains a last resort option for intractable epistaxis in recent guidelines and thus in the general perception of physicians treating this disease (23).

This narrative review aimed to provide an overview of posterior epistaxis etiologies, vascular anatomy, and a description of endovascular treatment, including its pre-procedural protocols, indications, and contraindications. We also aimed to discuss the most widely used techniques, potential complications and success rate.

## Materials and methods

We performed a systematic search in MEDLINE, Scopus, and Web of Science databases to identify articles relevant to our literature review. Our search query required the following keywords to be present in either the title or abstract: “epistaxis” OR “nosebleed”, AND “endovascular treatment”, “coils”, “embolic agents”, “embolization” or “endovascular procedures”. We initially identified articles throughout this search and two independent reviewers assessed the relevant articles by reviewing their titles and abstracts, eliminating those that met any of our exclusion criteria. Studies were excluded if they were 1) unrelated to epistaxis, 2) no endovascular treatment approach, 3) patients undergoing endovascular therapy for bleeding from other sources other than the nasal vasculature and 4) articles not written in English or Spanish. Conflicts were resolved by a senior researcher after discussion with reviewers. The remaining articles were thoroughly reviewed in their entirety to match our inclusion criteria. Articles were included if 1)included cases on posterior epistaxis, 2) articles about nasal and epistaxis related vascular anatomy, 3) articles that discussed endovascular treatment of epistaxis with a focus on technique, materials used, and possible complications. The remaining articles were completely read and relevant information was extracted to complete our narrative review.

## Results

A total of 1,435 articles were found after duplicate removal, after title and abstract screening only 383 were within the scope of our study. After full text screening only 181 articles met our inclusion criteria and were used to gather information for this literature review.

### Epidemiology

Epistaxis affects up to 60% of the world's population and is the most common otorhinolaryngological emergency and a frequent reason for emergency ENT consultations (5, 24). However, only 6% of cases require specialized medical attention as opposed to simple nasal packing done by any physician (5, 25). While posterior epistaxis accounts for a minority of epistaxis cases, it remains a life threatening situation that requires timely treatment (3, 4). Large studies have shown epistaxis to be equally frequent in males and females (26). Nonetheless, posterior epistaxis appears to be more common in elderly males, representing about 52.7% of cases (5, 10, 26).

### Etiology and comorbidities

Although most cases of posterior epistaxis are idiopathic (23), ranging from 60% to 88% of cases (6, 16, 20, 27), several other causes have been identified such as trauma, iatrogenic, coagulopathies, vascular tumors, vascular malformations (1, 15, 28). In most cases, posterior epistaxis is associated with underlying risk factors that alter hemostasis with the majority of cases being associated with hypertension, smoking, or the use of anticoagulation medications (6, 13, 16, 29). Other contributing factors include alcohol abuse (1, 7, 30), liver failure (20, 31), hypercholesterolemia (16, 31), and arteriosclerosis (7, 32).

Identifiable causes of posterior epistaxis can be categorized into local and systemic. Local causes include acute facial and nasal trauma, postoperative complications, tumor-induced factors, radiation-induced changes, and vascular malformations (16, 20, 23, 30, 31, 33). Systemic causes include coagulation disorders, hematological malignancies, hereditary hemorrhagic telangiectasia, among others (16, 23, 29).

### Nasal cavity vascular anatomy

**Figure 1:**
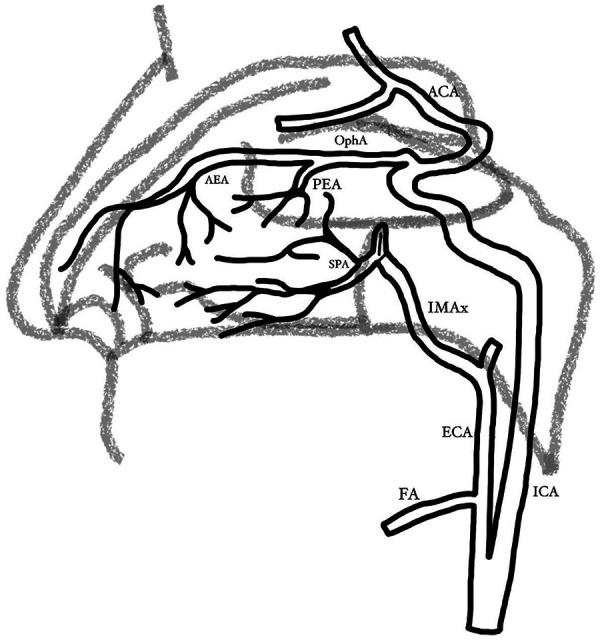
Vascular anatomy of nasal irrigation. ACA anterior cerebral artery, APA ascending pharyngeal, ECA external carotid artery, FA facial artery, ICA internal carotid artery, IMAx internal maxillary artery, artery, OphA ophthalmic artery, PEA posterior ethmoidal artery, SPA sphenopalatine artery, SPF sphenopalatine foramen.

The nose receives a rich blood supply through a complex arterial system involving multiple anastomoses (1, 17, 34). A thorough understanding of vascular anatomy is crucial for effective epistaxis management, minimizing risks, and ensuring successful surgical outcomes (1, 8, 11, 28, 35–37). The primary vascular supply to the nasal mucosa comes from the internal carotid artery (ICA) and external carotid artery (ECA) bilaterally, with their branches forming a dense vascular network in the nasal septum (Figure 1) (1, 8, 15). This includes the ethmoidal arteries from ophthalmic artery from ICA, the facial artery (FA), and the internal maxillary artery (IMAx) from the ECA (1, 8). Other branches of the ECA, such as the ascending pharyngeal artery (APA), are rarely involved in epistaxis pathophysiology (15, 38).

The vessels supplying the nasal mucosa have very little structural support, making them prone to bleeding from trauma or congestion due to allergies, infections, or dry environmental conditions (17, 34). Most cases of epistaxis arise from the anteroinferior nasal septum, known as the Little area, where the Kiesselbach plexus provides 80%–95% of the nasal blood supply (1, 5, 11, 16, 25). It is formed by the superior labial artery, the greater palatine artery, a branch of the sphenopalatine artery (SPA), and the anterior and posterior ethmoidal arteries (35). In the cases where the epistaxis originates from the posterior nasal cavity, Woodruff's plexus is the one mostly involved with the bleeding (5, 11, 36). It can be found over the posterior middle turbinate or within the posterosuperior part of the nasal septum (6, 36). It is formed by branches of the IMAx, such as the SPA, and a small contribution of the posterior ethmoidal artery (PEA) (11).

The IMAx is classified into three parts according to its relationship with the external pterygoid muscle. The proximal (mandibular) portion gives off five branches, including the middle meningeal and accessory meningeal arteries, which are considered dangerous for embolization (11). The pterygoid portion is in contact with the external pterygoid muscle. Finally, the terminal (pterygopalatine) portion forms a loop within the pterygopalatine fossa and gives rise to seven branches, with the greater palatine artery and the SPA being the most important for endovascular therapy in the treatment of epistaxis (1, 11). Within the pterygopalatine fossa, the SPA passes through the sphenopalatine foramen (SPF) along the lateral nasal wall (15, 16, 35, 36).

The SPA is the primary artery involved in intractable epistaxis (15, 16). As the main blood supply to the nasal cavity, its lateral branches supply the nasal turbinates, while its medial branches supply the nasal septum (11, 16). The crista ethmoidalis is a reliable landmark for locating the SPA (8). Notably, the SPA exhibits significant anatomical variability, with up to ten terminal branches (16). Surgeons must be aware of these variations, as differences in the SPA branches and foramen location may contribute to challenges in effective epistaxis control (6, 8, 36).

The nasal cavity also receives blood supply from the anterior and posterior ethmoidal arteries, branches of the ophthalmic artery (OphA), a branch of the ICA (8, 11, 35). The PEA travels through the posterior ethmoid canal to reach the posterior ethmoid cells (35). It irrigates the ethmoid sinuses, meninges, conchae, and nasal septum through various anastomoses with the facial and maxillary arteries (16). Additionally, the SPA communicates with the ethmoidal arteries via branches from the lacrimal artery of the OphA (11, 35, 39).

#### Dangerous communications

The complexity of the vascular network of the nose gives rise to numerous anastomoses. Potential connections between the ECA and ICA involved several arteries, including the artery of the foramen rotundum, the vidian artery, the middle meningeal artery, the accessory meningeal artery, the APA, the inferolateral trunk, and the meningohypophyseal trunk (5, 6, 15, 35, 39). Additionally, communications can occur between the FA, SPA, and OphA (5, 6, 15, 35), which are crucial to consider in order to ensure a safe embolization procedure (6).

Anastomoses between the SPA and branches of the ECA pose significant neurological risks. Off-target embolization in these areas can result in visual or central nervous system deficits (19). Additionally, the OphA may also have dangerous communications to the anterior deep temporal artery, and embolization of this region can lead to pterygoid muscle ischemia, which manifests as facial pain and trismus (6, 39, 40). Furthermore, potential connections between the ECA and cerebral or retinal circulations represent critical anastomoses that should be carefully identified and avoided during procedures (35, 39, 40).

It is crucial to be aware of dangerous anastomoses to the ICA in the pterygopalatine segment of the IMAx (i.e., the greater palatine and sphenopalatine arteries) (11, 35). These occur via the vidian artery (lateral segment) and the artery of the foramen rotundum (cavernous segment) (39). Another mention-worthy anastomosis is in the first section of the IMAX. Branches of the middle meningeal artery supply most dura mater and facial nerves, and its anterior branch communicates with the OphA (11, 16).

#### Anatomical variations

The high variability of the ECA system is a critical factor to consider during surgical interventions for posterior epistaxis. Unawareness of variations can lead to treatment failure. The SPA, due to its dominance in the nasal vasculature, has been studied extensively, as it is frequently involved in epistaxis (36). It is also important to mention the possibility of the complete absence of the SPA. The IMAx also exhibits significant variability, particularly along its third portion, which loops into the pterygopalatine fossa (36). Additionally, rare anatomical variants, such as the OphA arising from the middle meningeal artery rather than the ICA, must be recognized to avoid potentially devastating complications (40).

### Clinical presentation and diagnosis

In contrast to anterior epistaxis, posterior epistaxis is typically much more severe due to its origin from larger arterial vessels that lead into the nasopharynx (8, 41). This causes more substantial bleeding, which can be uncomfortable and may even induce nausea (8). Patients usually present precipitating factors, such as trauma or hypertension, and may exhibit signs of hemodynamic shock (7).

The need for imaging in epistaxis is largely dependent on the underlying etiology. It is typically unnecessary unless there is nasal or septal deformation (18). In cases where trauma is involved, an emergency CT scan is essential to identify maxilla-facial fractures, skull-base injuries, and intracranial injuries (27). Digital Subtraction Angiography (DSA) helps pinpoint the bleeding vessel on the ICA and ECA branches for intractable posterior epistaxis. Complete bilateral DSA of the internal and external carotid arteries (ICÁs and ECÁs) should be performed, with visualization in at least two planes that include the nasal cavity and orbital regions to reveal potential vascular variants (22, 28). Angiographic indicators of bleeding sources include vessel patency after prior surgical ligation, active extravasation, and enlarged or hypervascular mucosal staining (28). However, surgical ligation of the external carotid artery, while previously used to reduce perfusion, should be avoided whenever possible, as it eliminates endovascular access for subsequent treatment. Instead, an endovascular approach allows precise anatomical delineation, targeted occlusion of the bleeding vessel, and preservation of alternative vascular routes. After embolization an angiographic check is crucial to confirm hemostasis and exclude inadvertent vascular occlusion. Only after removal of the tamponade can the bleeding source be detected, if possible, this should be communicated by the ENT, since patients often arrive for vascular imaging with nasal tamponade in place.

### Conventional treatment options

When evaluating a patient with epistaxis, the primary focus is stabilization (1, 6, 8). Factors such as hypertension and the use of anticoagulant medications must be assessed, if visualization of the bleeding site cannot be achieved with anterior rhinoscopy, nasal endoscopy should be the next step, as it provides a clearer and magnified view of anterior structures, along with a direct view of the posterior part of the nose and the nasopharynx (5–7, 41). If the bleeding site can be determined through endoscopic equipment, decongestants and oxymetazoline nasal spray can be used. Electrocoagulation is preferred over silver nitrate because it is more effective and poses a lower risk of complications, including septal perforation, infection, rhinorrhea, and increased bleeding (1, 6–8, 10). If the patient is unstable and the bleeding site has yet to be identified, posterior packing is considered first-line treatment (1, 10, 36). The possible complications from this approach vary from posterior dislocation to staphylococcal toxic shock syndrome, thus making this a temporary fix that leads to a more invasive procedure such as surgical ligation (1, 6, 10, 36).

### Endovascular treatment

Digital subtraction angiography (DSA) with embolization offers several advantages, including precise anatomy, localization of the bleeding site, shorter procedural time, and preservation of the branches of the ECA or ICA. Endovascular approaches became prominent after Sokoloff et al. successfully performed a percutaneous embolization of the ipsilateral IMAx in two patients with Hereditary Hemorrhagic Telangiectasia (HHT) in 1974 (22). Two years later, Strother and Newton applied this technique in a series of patients, but it was not until 1980 that Merland et al. presented the first case series of 54 patients with severe epistaxis treated via endovascular embolization with a 97% success rate (42). Their findings supported the recommendation of embolization as a preferred method before opting for more invasive surgical interventions (43).

#### Protocols and technique

In emergency situations involving hemodynamically unstable patients with significant blood loss, extensive diagnostic testing should not delay treatment. Hemoglobin level is the most important parameter, as it determines the need for immediate endovascular intervention. When the status of the patient allows, an initial assessment should be carried out, including checks for hemoglobin, platelet count, coagulation studies, INR, creatinine and thyroid-stimulating hormone (TSH) (24). To ensure the safety of endovascular embolization, a diagnostic DSA with adequate visualization of the ICA and ECA should be performed first, with particular attention to identifying dangerous extracranial-intracranial anastomoses. This initial angiographic assessment will influence the embolization strategy, including the feasibility of distal vs. proximal embolization, the need for unilateral or bilateral treatment, and the selection of embolic materials (22, 28). Angiography should be done with a standard femoral or radial approach and should include images in the arterial phase and in the tissue phase (16, 33). In modern practice, guiding catheters (0.066 or 0.079″) are used rather than diagnostic catheters as they have a larger lumen that allow simultaneous vessel control during embolization, even when a microcatheter is in place (24, 42). This procedure serves two purposes: to identify an underlying disease as the etiology of epistaxis and to determine the patient's anatomy preoperatively, thus gaining awareness of any dangerous anastomoses (39). These procedures should therefore be performed only by interventional neurosurgeons, neurologists or radiologists with extensive experience in neurovascular anatomy.Tseng et al. (27) suggest that the presence of choroidal blush on the ECA signals collateral blood flow to the eye, which should be monitored to prevent complications. In cases of bilateral bleeding, angiography should be performed bilaterally (13, 29). Some authors also recommend the use of cerebral and supraoptic vessel CT angiography to further identify the source of bleeding (24).

General anesthesia is typically used to protect the airway in patients with active or massive intraoral blood accumulation (43). Local anesthesia may be used in cooperative patients with minor active bleeding or when anesthesia is contraindicated (27, 36). Cohen et al. (44) presented a retrospective study in which nearly three-quarters of the patients were successfully treated with local anesthesia and conscious sedation. General anesthesia remains necessary for those who require airway protection or movement control.

Authors describe various endovascular approaches for epistaxis. Frank et al. (21) outline the different approaches by targeted vessel, which include: 1) embolization of the ipsilateral IMAx and SPA, 2) ipsilateral and contralateral IMAx/SPA, and 3) ipsilateral IMAx/SPA in combination with the ipsilateral or contralateral FA. The choice of vascular access, femoral or radial, depends on operator preference and patient anatomy. Guiding catheters of 5F or 0.066 or 0.079″ (Envoy or Simmons) are typically used to achieve stable access. Seldingeŕs technique is the most widely used, with an approach through the right femoral artery, although recently, the use of radial access has become widespread for neuro-interventional procedures (37). For elderly patients, a 0.079″ Simmons 2-guide catheter is preferred (43). A bolus of 5,000 IU heparin is administered before the procedure, followed by 1,000 IU per hour continuous infusion for maintenance. Recently, some authors have described a feasible approach to access the IMAx through an open retrograde method via the superficial temporal artery (45). Access to the IMAx via the ECA and its distal branches SPA and DPA may be performed using various microcatheter systems, individualized for each patient depending on anatomic factors such as the variable angulation of the ECA- IMAx junction, the tortuosity of the pterygopalatine segment, and the small caliber of distal branches like the SPA and DPA. The operator must determine the catheter stability, navigability and safety and therefore choose the optimal setup. Commonly used systems include microcatheters such as the Echelon, Headway Duo, or Marathon paired with their appropriate microguidewires.

Several materials have been described in the literature for endovascular treatment, including polyvinyl alcohol particles, coils, and liquid embolizing agents (absorbable hemostatic sponges, cyanoacrylate adhesives, and ethylene-vinyl alcohol derivatives) (6, 12, 16). The selection of endovascular material is primarily based on the underlying cause of the epistaxis (16). For posterior epistaxis embolization, microparticles of 250–350 µm are preferred (2, 32, 42, 46). Smaller particles (150–250 µm) are generally avoided, given they may cause tissue necrosis and pass through unrecognized anastomoses between the ECA and ICA or the OphA, causing potential complications such as stroke or blindness (29, 33, 39). Robinson et al. recommended the use of smaller particles when accessing the distal part of the IMAx (33). On the other hand, Reyre et al. mention the use of non-resorbable microparticles with a 500 µm diameter as extremely effective (17). The choice of embolic agent should therefore be individualized, balancing distal penetration and hemostatic efficacy against the risk of off-target embolization through unrecognized anastomoses. The decision between unilateral and bilateral embolization is an important technical consideration. Unilateral embolization may be sufficient when a dominant bleeding vessel is identified angiographically. However, bilateral embolization may be required in cases of extensive collateralization, bilateral bleeding, or recurrent epistaxis. While bilateral treatment may improve hemostatic durability, it also increases the risk of ischemic complications and should be performed cautiously, guided strictly by angiographic findings.

Coils are also a viable option and are available in “flushable” or controlled-release varieties. They should be placed as distally as possible, ensuring contact with the hemorrhagic area (16). The use of coils is mostly avoided since they only allow proximal embolization and, in case of recurrence, prevent further reintervention (12, 16, 28). However, in the presence of dangerous anastomoses or large arterial wall defects, embolization with coils is recommended. This may lead to more proximal and less effective embolization, but it is still necessary to stop the bleeding (12, 28). Cyanoacrylate glues are challenging to use and require operators with specialized training (16). In contrast, embolization with ethylene-vinyl alcohol derivatives is simpler and carries a lower risk of off-target embolization (16). Techniques where different materials are used in combination have been described (12).

#### Indications and contraindications

Endovascular treatment of epistaxis can be used in various circumstances. Primary embolization is appropriately indicated in severe epistaxis with hemorrhagic shock or in cases where surgery may not be feasible (6). It is also used for idiopathic, refractory, or secondary epistaxis related to HHT or tumors (16). Hofmanns et al. emphasized selective, anatomy-guided embolization to optimize hemostasis and reduce ischemic risk, while Koskinas et al. highlighted tailored distal vs. proximal occlusion based on collateral flow. Many limitations have been described for an endovascular approach, though most of them are considered relative (6). Some of these relative contraindications include severe arteriosclerosis, high-flow anastomosis with the ICA, tortuous artery, allergy to contrast material, or any involvement of ICA (4).

#### Complications

Endovascular treatment may present different complications, which can be classified into minor and major categories (Table 1) (13, 18, 19, 23). It is important to mention that major complications are rare, accounting for only 2% of the overall complications.

**Table 1:** Classification of complications in endovascular treatment of epistaxis.

Minor	Major
Facial pain or numbness Headache Trismus Access point pain Hematoma due to cannulation	Ischemic stroke Blindness Seizures Facial paralysis Soft tissue necrosis

#### Success rate

Different case series report various success rates of endovascular treatment for epistaxis depending on the artery embolized (Table 2). Some authors have emphasized the correlation between the number of embolized arteries and an increased success rate (4).

**Table 2:** Success rate of endovascular treatment for posterior epistaxis.

Authors	No. Cases	Artery	Materials	Success Rate
Vizzuso et al. (47)	32 patients	SPA	250–355 µm polyvinyl alcohol particles	100%
El Naamani et al. (9)	35 patients	IMA (86.4%), FA (13.6%)	Onyx (45%), particles (20), or combination of both (35%)	97.5%
Lelegren et al. (48)	33 patients	SPA	–	92.6%
Franke et al. (24)	123 patients	SPA, IMA	Microparticles and coils	95.1%
Robinson et al. (33)	59 patients	Bilateral distal IMA, unilateral or bilateral FA	Polyvinyl alcohol particles	97.1%
Wehrli et al. (49)	19 patients	IMA	–	74%
Vitek (50)	30 patients	IFA	–	97%
Oguni et al. (13)	37 patients	IFA ECA	–	94.6%
Siniluoto et al. (51)	31 patients	–	–	75%
Elahi et al. (52)	57 patients	–	–	86%

ECA, External Carotid Artery; IFA, Ipsilateral Facial Artery; FA, facial artery; IMA, internal maxillary artery; SPA, Sphenopalatine Artery.

### Proposed clinical decision-making algorithm for posterior epistaxis

The management of posterior epistaxis requires an individualized, multidisciplinary approach that takes into account bleeding severity, patient comorbidities, vascular anatomy seen upon DSA, and professional expertise. Based on the available literature and accumulated clinical experience, a stepwise decision-making algorithm can be proposed to guide treatment selection. Initial management should focus highly on hemodynamic stabilization and correction of reversible factors such as hypertension and coagulopathy. When conservative measures fail or bleeding is severe, definitive intervention is required.

Endovascular embolization should be preferred in patients with severe or recurrent posterior epistaxis who are poor surgical candidates, have failed prior surgical ligation, present with bilateral or multifocal bleeding, or require precise localization of the bleeding source. Embolization is easier when angiography reveals identifiable bleeding vessels without high-risk extracranial–intracranial anastomoses.

Surgical management, most commonly endoscopic artery ligation, may be preferred in patients with adequate surgical anatomy, absence of significant comorbidities, and when rapid endoscopic identification of the bleeding source is possible. Surgery may also be preferred in centers without immediate endovascular experts. Combined approaches may be appropriate in complex cases, such as recurrent epistaxis after prior embolization or surgery, persistent bleeding due to extensive collateralization, or tumor-related epistaxis requiring both devascularization and surgical control.

Endovascular embolization should be avoided in the presence of uncontrolled high-flow anastomoses between the external and internal carotid systems seen on DSA, extensive ICA involvement, or when superselective catheterization distal to dangerous anastomoses cannot be safely achieved. In such scenarios, surgical alternatives or conservative measures may be safer.

**Figure 2:**
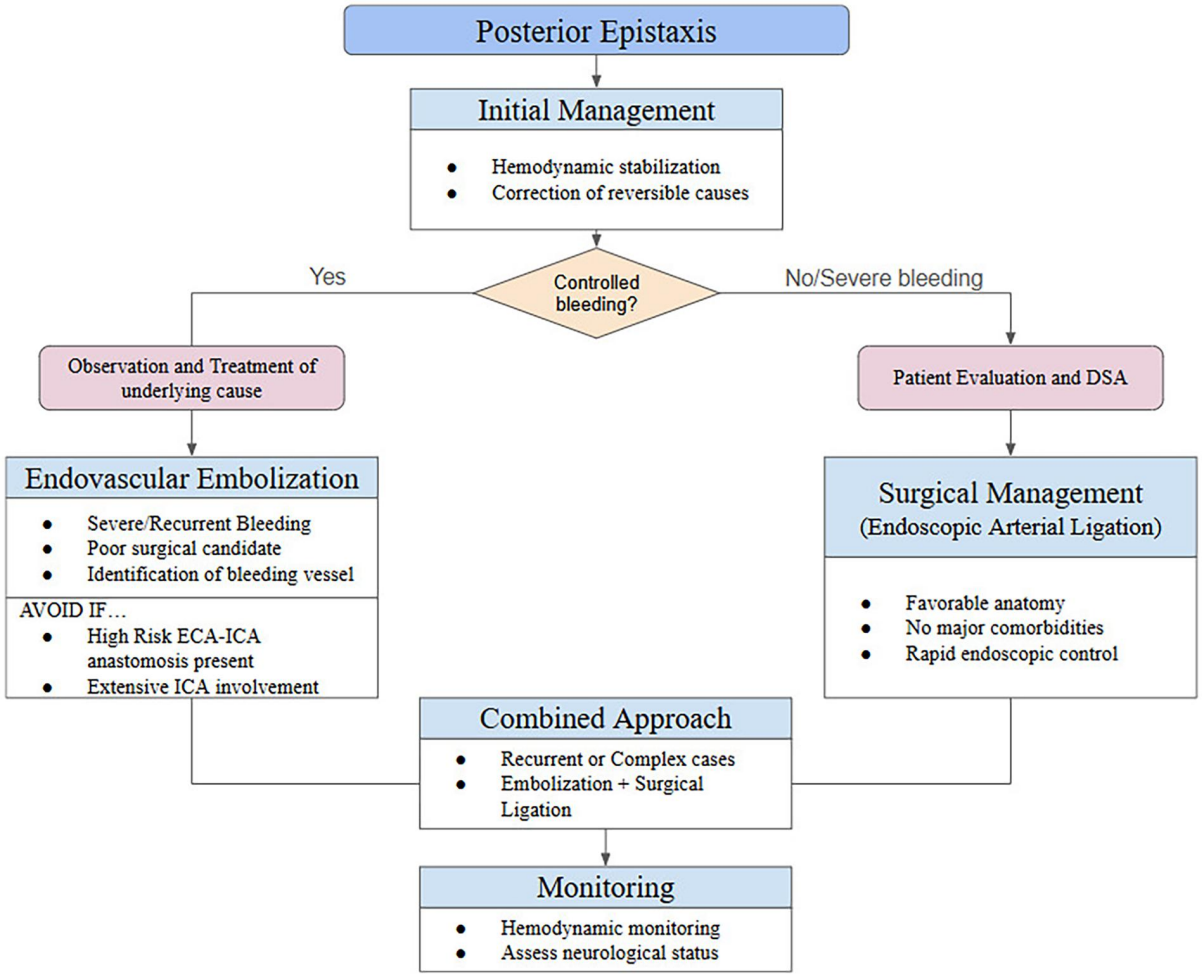
Proposed treatment algorithm for the treatment of posterior epistaxis.

A proposed treatment algorithm summarizing this decision-making process is presented in Figure 2, aiming to provide practical guidance for clinicians involved in the management of posterior epistaxis.

## Discussion

The wide range of posterior epistaxis severity and causes, as well as the complex vascular anatomy, highlight the importance of an individualized approach when determining treatment options for each patient. Identifying the most appropriate course of treatment is especially crucial in cases of intractable epistaxis. Since no treatment is without risks, it's essential to carefully balance the patient's clinical state and prognosis with the potential benefits and risks of different treatment modalities (Table 3).

**Table 3:** Possible complications of epistaxis treatments.

Treatment Modality	Possible Complications
Cauterization	Septal perforation, infection, rhinorrhea, and increased bleeding. (7, 8, 41)
Nasal Packing	Posterior dislocation, foreign body reaction, eustachian tube dysfunction, sinusitis, infections (e.g., otitis media, staphylococcal toxic shock syndrome, pneumonia), hypoxia, hypoventilation, aspiration, septal hematomas, abscesses, neurogenic syncope, ulcerations, mucosal necrosis, septal perforation, myocardial infarction, and cerebral ischemia. (1, 10, 17, 38)
Surgical Ligation	Recurrence of bleeding, nasal dryness crusting, palatal numbness, blindness, sinusitis, infection, decreased lacrimation, injury to the infraorbital nerve, oroantral fistula, and epiphora. (1, 6, 10)
Endovascular Treatment	Facial pain or numbness, headache, trismus, access point pain, hematoma due to cannulation, ischemic stroke, blindness, seizures, facial paralysis, trismus, soft tissue necrosis, and swelling of the cheek. (13, 17, 19, 23)

Embolization can be used as a subsequent treatment when other treatment modalities fail. However, it can also serve as an alternative to prolonged nasal packing or ligation surgery (6). Embolization and surgery are constantly compared in the literature regarding their success and failure rates, complication rates, contraindications, costs, material availability, and comorbidities (6). The advantages of each procedure have been well documented in literature (Table 4) (5, 6, 20).

**Table 4:** Advantages between endovascular treatment vs. Endoscopic Ligation.

Endovascular Treatment	Endoscopic Ligation
Shorter hospital stays. Immediate control of epistaxis. Possibility of local anesthesia.	Lower costs. Shorter hospital stays. Need for less reintervention.

In endovascular treatment, superior skill and detailed knowledge of the vessels involved, along with their multiple anastomoses, are crucial for a safe and reliable procedure (8, 11, 35, 39). Additionally, a physician performing a transarterial embolization must have a thorough understanding of the potential dangerous anastomoses in the nasal vasculature (Table 5) (11, 16, 35). For this reason, it is imperative that the primary operator should always be a neuro-interventionalist with experience in cerebral and craniofacial circulation. Some connections between the ICA and ECA systems may open under increased pressure, allowing embolic material to pass from the extracranial circulation into the anterior intracranial circulation (11, 16, 35, 39). Identifying and steering clear of these ‘dangerous anastomoses’ is a critical step in preventing inadvertent embolization (16, 25, 28, 32, 34, 39). Pre-embolization DSA of the ECA and ICA is crucial for this purpose (28). If anastomoses are found, embolization should be done as distally as possible from the anastomosis or not done at all, thus ensuring patient safety and avoiding complications (33).

**Table 5:** Summary of dangerous anastomoses and their possible complications,.

Possible Complication	Anastomoses
Stroke	IMAx & Middle meningeal artery. IMAx & Accessory meningeal artery. IMAx & Vidian artery. IMAx & Artery of the foramen rotundum
Blindness	IMAx & Lacrimal artery (OphA). ECA & Retinal arteries. OphA & Anterior deep temporal artery
Facial Pain	OphA & Anterior deep temporal artery
Trismus	OphA & Anterior deep temporal artery

ECA, External Carotid Artery; IMAx, Internal Maxillary Artery; OphA, Ophthalmic Artery.

The development of newer, less invasive techniques that allow for selective control of bleeding makes it especially relevant to consider the complexity and potential variability of the anatomical vascular supply. The selection of embolic material can impact rebleeding rates. In their meta-analysis, Hoffman et al. reported that the most frequently used embolic agents were spherical particles, followed by coils and Onyx, an ethylene-vinyl alcohol derivative (23). Coils offer the advantage of controlled deployment and a lower risk of penetrating the OphA or intracranial arteries (12, 16, 23). However, they have been associated with a higher risk of rebleeding in some studies (23). On the other side, small particles run the risk of passing through unrecognized anastomoses, causing tissue necrosis (29, 32). Some embolic agents, like absorbable hemostatic sponges, dissolve in hours, allowing for temporal devascularization (12). To avoid any potential complications of endovascular treatment, it is important to remember that it should always be preceded by a comprehensive angiography of the vascular system (5). Despite the potential complications, endovascular embolization in cases of posterior epistaxis has a high rate of immediate post-intervention success, according to some studies, with a relatively low rebleeding rate of 16.4% and an immediate control rate of up to 92.6% (6, 20, 23).

## Limitations

Firstly, our research was limited to three databases (MEDLINE, Scopus, and Web of Science) and included papers written only in English or Spanish, aiming to ensure a comprehensive understanding of the articles selected. Given the absence of standardized guidelines, a higher level of evidence is needed to support this work. Finally, it is important to note that this study is a narrative review article, not a systematic review, which inherently carries different limitations.

## Conclusion

Endovascular treatment is a viable and safe option in cases of posterior epistaxis and should not be reserved solely as a last resort. It can have high success rates with low rates of major complications. Even so, given the complex nasal vascularity and the potentially dangerous anastomoses, a thorough understanding of the anatomy and previous imaging studies are imperative for the safety and effectiveness of this procedure. Ongoing advances in preoperative imaging, as well as endovascular techniques, embolizing agents, and catheter technology, continue to enhance the safety and reliability of this treatment method. However, in the absence of standardized guidelines, we emphasize the crucial need for homogenizing criteria among the different specialties involved in the management of posterior epistaxis. Large-scale, controlled, prospective studies are essential to establish, regulate, and generate these much-needed guidelines.

## Data Availability

The original contributions presented in the study are included in the article/Supplementary Material, further inquiries can be directed to the corresponding author.
